# Potential Neurotoxic Effects of Glioblastoma-Derived Exosomes in Primary Cultures of Cerebellar Neurons via Oxidant Stress and Glutathione Depletion

**DOI:** 10.3390/antiox11071225

**Published:** 2022-06-23

**Authors:** Sidika Genc, Manuela Pennisi, Yesim Yeni, Serkan Yildirim, Giuseppe Gattuso, Meric A. Altinoz, Ali Taghizadehghalehjoughi, Ismail Bolat, Aristidis Tsatsakis, Ahmet Hacımüftüoğlu, Luca Falzone

**Affiliations:** 1 Department of Pharmacology, Faculty of Medicine, Bilecik Seyh Edebali University, Bilecik 11230, Turkey; ssidika@atauni.edu.tr; 2 Department of Biomedical and Biotechnological Sciences, University of Catania, 95123 Catania, Italy; peppeg9305@gmail.com; 3 Department of Medical Pharmacology, Faculty of Medicine, Ataturk University, Erzurum 25240, Turkey; yesim.yeni@atauni.edu.tr; 4 Department of Pathology, Faculty of Veterinary Medicine, Ataturk University, Erzurum 25240, Turkey; syildirim@atauni.edu.tr (S.Y.); ismail.bolat@atauni.edu.tr (I.B.); ahmeth@atauni.edu.tr (A.H.); 5 Department of Biochemistry, Acibadem Mehmet Ali Aydinlar University, İstanbul 34684, Turkey; maltinoz@gmail.com; 6 Department of Forensic Sciences and Toxicology, Faculty of Medicine, University of Crete, 71003 Heraklion, Greece; tsatsaka@uoc.gr; 7 Federal Scientific Center of Hygiene F. F. Erisman, 2 Semashko Street, Mytishchi 141014, Moscow, Russia; 8 Epidemiology and Biostatistics Unit, National Cancer Institute-IRCCS Fondazione G. Pascale, 80131 Naples, Italy

**Keywords:** glioblastoma multiforme, exosome, cerebellum, oxidative stress, glutathione, neurotoxicity, neuro-oncology

## Abstract

High-grade gliomas are the most fatal brain tumors. Grade 4 gliomas are called glioblastoma multiforme (GBM), which are associated with the poorest survival and a 5-year survival rate of less than 4%. Many patients with GBM developed concomitant cognitive dysfunctions and epilepsy. Although the cognitive decline is well defined in glioblastomas, the neurotoxic factors underlying this pathology are not well understood in GBM patients. In this study, we aimed to investigate whether GBM-derived exosomes play a role in neuronal toxicity. For this purpose, exosomes obtained from T98G and U373 GBM cells were applied to primary neuron culture at different concentrations. Subsequently, MTT, LDH, GSH, TAS, and TOS tests were performed. Both GBM-derived exosomes induced a dose-dependent and statistically significant increase of LDH release in cerebellar neurons. MTT assay revealed as both T98G and U373 GBM-derived exosomes induced dose-dependent neurotoxic effects in cerebellar neurons. To the best of our knowledge, this study is the first study demonstrating the toxic potential of GBM-derived exosomes to primary neurons, which may explain the peritumoral edema and cognitive decline in GBM patients.

## 1. Introduction

Gliomas are the most lethal tumors affecting the brain. Among these, glioblastoma multiforme (GBM) is the most advanced brain tumor and it is associated with the poorest survival rate, with a 5-year survival rate of less than <4% [1]. GBM accounts for about 80% of primary brain malignancies and influences more than 17,000 patients every year in the USA [1]. For many patients with GBM, deficits in cognitive functioning and epilepsy are part of the disease process [2,3]. The decline in cognitive functioning is an important clinical symptom useful to predict the prognosis of glioma patients and may be the first indicator of disease recurrence [4,5]. In an animal model, Wang et al. investigated the cognitive functions and the role of glutamate and synaptic plasticity in C6 glioma-bearing rats in which tumor cells were injected into the right caudate putamen nucleus [2]. C6 glioma-bearing rats exerted significant perturbations of short-term potentiation and long-term potentiation associated with memory [2]. Glutamate level concentration and glutamate/γ-aminobutyric acid ratio were significantly increased in contralateral hippocampus that was not implanted with tumor cells, indicating impairment of synaptic transmissions due to factors not limited to tumor infiltration [2]. Neuro-inflammation triggers local hypertension and edema, all of which could injure the dendritic spines. Indeed, reduced dendritic spine density, disarray of microtubules, and swollen dendrites are classical features of peritumoral edema in brain malignancies [6]. Vice versa, anti-inflammatory nanoparticles increase the synaptic plasticity in hippocampus and reduce the extent of cognitive decline induced by intracranial C6 glioma as shown in both the open field and the novel object recognition tests [6].

Despite cognitive decline being well described in GBM, the underlining factors of this pathology—independent from brain radiotherapy and chemotherapy and apart from neuroinflammation—are not well understood. It is very likely that GBM cells release factors that may exert neurotoxic effects causing cognitive impairments [7,8,9]. Among these factors, glutamate release by primary brain tumors has been associated with epileptic seizures [10]. More recently, Wei and colleagues also demonstrated that human glioma stem cells release microvesicles and exosomes containing coding and non-coding RNA molecules which interact with the surrounding neuronal structure [11]. In brain tissue, these vesicles can drive the differentiation of neural stem cells to astrocytes creating a pro-inflammatory microenvironment or induce cognitive decline through multiple mechanisms [12,13].

No studies on the neurotoxic effects of GBM in cerebellum exist and several GBM patients exhibit neurological disorders and motor dysfunction related to the alteration of cerebellar structures due to the presence of GBM or the adverse effects of GBM-related treatments [14,15,16,17]; therefore, the present study was aimed to explore whether GBM-derived exosomes may play a role in neuronal toxicity in primary cerebellar neurons. For this purpose, T98G and U373 GBM cell lines were used as these two in vitro models are widely used in GBM functional studies.

Notably, exosomes—which were first described by Johnstone et al. in 1987 [18]—comprise vesicles of endosomal membrane with diameters of ~40 to 150 nm, which develop from the late endosomes via inward budding of endosomal membranes, forming intracellular multivesicular endosomes [18]. Exosome pools are packed in the multivesicular endosomes and released into the extracellular compartment after the fusion of the multivesicular endosomes with the plasma membrane [19]. Exosomes could convey genetic material (RNA and DNA), protein, and lipids to juxtaposing cells or even to cells far away from their origin [20]. Despite that many hundreds, or even thousands, of proteins could be extracted from exosomes, their main protein markers include Alix (programmed cell death 6-interacting protein), CHC1 (Clathrin heavy chain 1), CD63 (lysosomal-associated membrane protein 3), TSG101 (Tumor susceptibility gene 101 protein), and flotilin-1 [20]. Most of the current literature on the pathobiology of cancer cell-derived extracellular vesicles focuses on the fact that these exosomes convey oncogenic material to their vicinity or even far from their origin to increase tumorigenicity or even transform other cells [21]. On the other hand, it was demonstrated that cancer cells release extracellular vesicles which contain tumor suppressor and proapoptotic proteins including P53, DKK4, PTEN, and maspin, among others [21]. This feature is attributed to a putative mechanism elicited by cancer cells to “deport” proapoptotic, growth-suppressive and senescence-inducing factors from their cytoplasm [21]. However, the release of such factors by malignant cells may also serve to induce cell death in their vicinity to foster tumor invasion.

## 2. Materials and Methods

### 2.1. Glioblastoma Cell Lines and Primary Neuron Cell Culture

The GBM cell lines T98G and U373 were kindly provided by the researchers of the department of veterinary pharmacology and toxicology of Ataturk University (Erzurum, Turkey). Briefly, the cells were resuspended in fresh medium (Dulbecco’s modified eagle medium (DMEM), Fetal bovine serum (FBS; 15%), and antibiotic suspension 1% (penicillin, streptomycin, and amphotericin B). Then, the cells were plated into 150 cm^2^ cell culture flasks (Corning, Corning, NY, USA) and cultured in a CO_2_ incubator under appropriate conditions (5% CO_2_; 37 °C).

Sprague Dawley newborn rats born within 24 h were used to obtain cerebellum neurons in order to test the effects of glioblastoma-derived exosomes. Briefly, after sedation, the rats were rapidly decapitated, brain tissue was collected, and cerebellar tissue was isolated with the help of a scalpel. Then, a single cell suspension was obtained with Trypsin-Ethylenediaminetetraacetic acid (EDTA) (0.25% trypsin–0.02% EDTA) dissociation. The cells were then centrifuged at 1200 rpm for 5 min. Then the cells were resuspended in an appropriate medium (88% NBM (Neuro-basal medium, Gibco, Waltham, MA, USA) with 10% FBS (Fetal bovine solution, Gibco, USA), 2% B-27 (Thermo Fisher, Bremen, Germany), 0.1% antibiotic (Penicillin–Streptomycin), and amphotericin B (Thermo Fisher, Germany). The cells were incubated at 5% CO_2_ at 37 °C for 10 days by changing the medium every 3 days.

### 2.2. Exosome Isolation and Primary Neuron Cell Treatment

To obtain GBM-derived exosomes, T98G and U373 cell lines were plated in T75 cm^2^ flasks. After 70% confluency in 150 cm^2^ cell culture flasks (Corning, USA), 25 mL of complete medium was added. Subsequently, the Total Exosome Isolation Reagent (Invitrogen™—Cat. 4478359, Waltham, MA, USA) protocol for the effective isolation of exosomes was used as previously described [22,23]. Briefly, conditioned medium and reagent (2:1) were mixed and incubated at 2 °C to 8 °C overnight. After incubation, the samples were centrifuged at 10,000× *g* for 1 h at 2 °C to 8 °C in order to pull down the exosomes at the bottom of the tube.

A wide range of exosome concentrations was used as follows: 1–5–10–50–100 µg/mL. Such concentrations were selected on the basis of similar studies reported in literature [24,25,26,27]. In detail, exosomes at different concentrations were added into 96 well plates, containing primary neuron cells at 85% confluence to assess the toxicity of GBM-derived exosomes. After 24 h of incubation (at 37 °C, 5% CO_2_ and 95% humidity), MTT assay and immunohistochemical staining were performed and TAS, TOS, GSH and LDH levels were evaluated. Untreated cells were used as controls for biochemical and immunofluorescence evaluations.

### 2.3. MTT Assay

After 24 h of treatment with the GBM-derived exosomes, 10 μL of MTT solution (1:10) was added to each well plate. After 4 h of incubation, 100 μL of DMSO solution was added to all wells. The optical density of the dissolved formazan crystals was determined at a wavelength of 570 nm by using the Multiskan™ GO Microplate Spectrophotometer reader.

### 2.4. Total Oxidant Status (TOS) and Total Antioxidant Status (TAS)

TOS and TAS (Rel Assay Diagnostics^®^ Company, Gaziantep, Turkey) were determined with spectrophotometry (Multiskan™ GO Microplate Spectrophotometer reader, Waltham, MA, USA). The chromogenic xylenol orange was used to evaluate TOS levels as under acid conditions, the oxidizing potential of samples can oxidize Fe^2+^ to Fe^3+^ which binds xylenol orange and convert it into a blue-purple complex. To determine the TOS levels, 75 µL of cell supernatant and 500 µL of reactive 1 solution were added to each well and then read at 590 nm. For the second reading, 25 µL of reactive 2 solution was added to the wells and the absorbance was read at 590 nm (TOS = Δexample/ΔST2 × 20). To evaluate the TAS levels, ABTS (2,2′-azino-bis(3-ethylbenzothiazoline-6-sulfonic acid), a chromogenic substance that can be converted by oxidizing agent into its radical monocation form ABTS+ (colored), was used. More in detail the TAS of the samples was determined and calculated by measuring the absorbance of ABTS+ at 660 nm. Trolox is an analog of Vitamin E and has a similar antioxidant state to that of Vitamin E. Trolox is used as a reference substance for total antioxidant status. During this assay, 500 µL of reactive 1 solution was added to the wells containing 30 µL of cell supernatant. The first read was done at 660 nm. For the second read, 75 µL of reactive 2 solution was added in the wells and absorbance was read at 660 nm (TAS = (ΔST1 − Δexample)/(ΔST1 − ΔST2)).

### 2.5. Lactate Dehydrogenase (LDH) Assay 

LDH assay test was performed using a commercially available test kit from Cayman Chemical Co., Ltd., (Ann Arbor, MI, USA). Briefly, the cell culture medium was centrifuged at 400× *g* for 5 min at room temperature. A volume of 100 µL of the supernatant was added to 100 µL of the reaction solution (LDH Assay Buffer, LDH Substrate Mix) and incubated with gentle shaking on an orbital shaker for 30 min at room temperature. Finally, the absorbance was read at 490 nm wavelength.

### 2.6. Glutathione (GSH) Assay

The glutathione (GSH) measurement is based on the principle of glutathione reacting with dithionitrobenzoic acid (DTNB) to produce thio-nitrobenzoic acid and glutathione disulfide. Optical density (OD) is measured spectrophotometrically at 450 nm ± 2 nm wavelength. The GSH concentration in the samples tested is calculated by comparing the OD of the samples with the standard curve.

GSH + DTNB → GSSH + TNB

For GSH determination, Elabscience kit (Houston, TX, USA) was employed and the intensity of the yellow color formed by nitrobenzoic acid was read in a spectrophotometer at 450 nm wavelength.

### 2.7. Statistical Analyses

Statistical analyses were performed using one-way analysis of variance (ANOVA) with Tukey’s HSD for post-hoc comparisons using the SPSS 22.0 software and the results were presented as median ± SEM. A *p*-value of *p* < 0.05 was considered statistically significant. All the experiments were performed in triplicate. 8-OHdG fluorescent signal was observed in five different microscopic fields.

## 3. Results

### 3.1. Alteration of Glutathione (GSH) Levels in Cerebellar Neurons after GBM-Derived Exosomes Treatment

**Figure 1:**
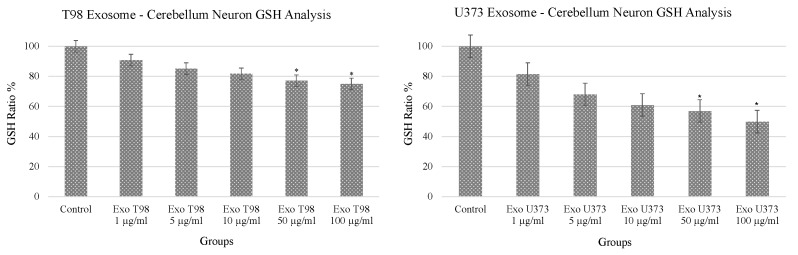
Significant reduction of glutathione levels in cerebellar neurons treated with T98G- and U373-derived exosomes. ANOVA One-Way * *p* < 0.05.

The analysis of GSH revealed a dose-dependent reduction of GSH levels in cerebellar neurons after the exposure to both T98G and U373 GBM-derived exosomes. In particular, statistical differences in GSH levels were observed in cerebellar neurons treated with 50 and 100 µg/mL of exosomes. Of note, GSH depletion was more prominent in cells exposed to U373 GBM-derived exosomes compared to T98G-derived exosomes showing almost 50% reduction of GSH compared to non-treated cells (Figure 1). 

### 3.2. Lactate Dehydrogenase (LDH) Levels in Cerebellar Neurons after GBM-Derived Exosomes Treatment

**Figure 2:**
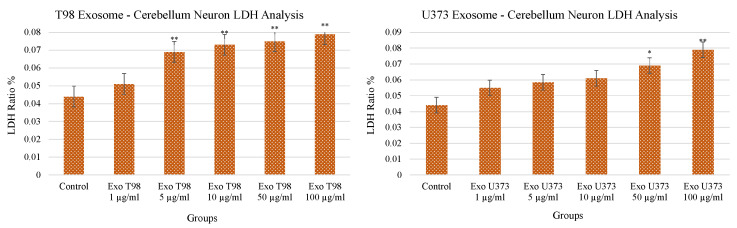
Significant increment of LDH levels in cerebellar neurons treated with T98G- and U373-derived exosomes. ANOVA One-Way * *p* < 0.05; ** *p* < 0.01.

Both T98G- and U373-derived exosomes induced a dose-dependent and statistically significant increase of LDH release in the culture medium of cerebellar neurons. LDH levels increased in a dose-dependent manner mainly in cerebellar neurons exposed to T98G-derived exosomes compared to those treated with U373 exosomes (*p* < 0.01) at low concentrations (≥5 µg/mL). In cerebellar neurons exposed to U373-derived exosomes, the statistical significance (*p* < 0.05) was reached at a concentration of 50 µg/mL and occurred more evidently at 100 µg/mL (*p* < 0.01) (Figure 2). The increased levels of LDH demonstrated the harmful potential of GBM-derived exosomes able to induce damages in cerebellar neurons and, consequently, neuron cell death.

### 3.3. Treatment with GBM-Derived Exosomes Reduces Cell Viability of Cerebellar Neurons In Vitro

**Figure 3:**
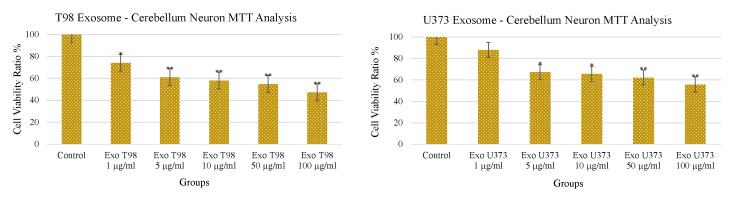
Reduction of cerebellar neuron viability after treatment with T98G- and U373-derived exosomes. * *p* < 0.05; ** *p* < 0.01.

The treatment with increasing doses of T98G- and U373-derived exosomes induced a dose-dependent and statistically significant depletion of MTT activity in cerebellar neurons. As observed for the LDH levels, T98G-derived exosomes also induced a more pronounced depletion of MTT activity. An amount of 1 µg/mL of T98G-derived exosomes depleted MTT with a statistically significant difference of *p* < 0.05. Higher dosages ≥ 5 µg/mL depleted MTT in a more significant manner (*p* < 0.01). In cerebellar neurons exposed to U373-derived exosomes, a significant reduction of cell viability (*p* < 0.05) was obtained at 5 and 10 µg/mL while a more consistent reduction of the MTT activity was observed at 50 and 100 µg/mL of exosomes (*p* < 0.01) (Figure 3).

### 3.4. Total Antioxidant Status (TAS) and Total Oxidant Status (TOS) in Cerebellar Neurons Treated with T98G and U373 GBM-Derived Exosomes

**Figure 4:**
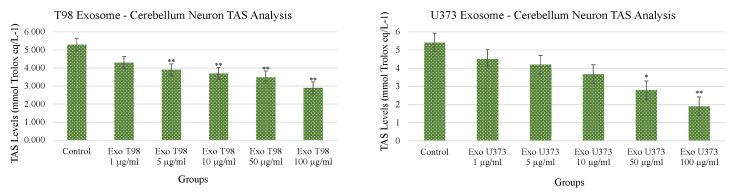
Reduction of Total Antioxidant Status of cerebellar neurons treated with T98G- and U373-derived exosomes. ANOVA One-Way * *p* < 0.05; ** *p* < 0.01.

Both T98G- and U373-derived exosomes induced dose-dependent and statistically significant reduction of TAS. The extent of TAS depletion occurred with higher significance when cerebellar neurons were exposed to T98G-derived exosomes. At concentrations ≥5 µg/mL of T98G-derived exosomes, TAS depletion occurred with a statistical significance of *p* < 0.01. In cerebellar neurons exposed to U373-derived exosomes, the statistical significances of TAS depletions were *p* < 0.05 and *p* < 0.01 for exosome concentrations of 50 and 100 µg/mL, respectively (Figure 4).

**Figure 5:**
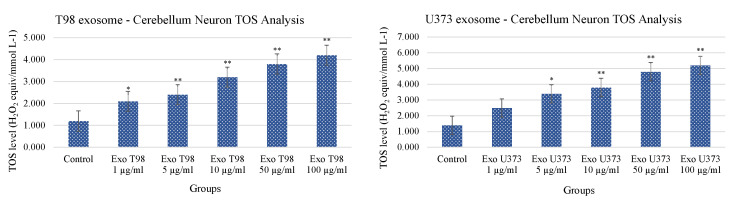
Increment of Total Oxidant Status of cerebellar neurons treated with T98G- and U373-derived exosomes. ANOVA One-Way * *p* < 0.05; ** *p* < 0.01.

Conversely to what was observed for TAS levels, the analysis of TOS revealed that cerebellar neurons treated with T98G- and U373-derived exosomes showed a statistical increase of TOS levels with a dose-dependent trend. The extent of TOS increase was more marked in cerebellar neurons exposed to T98G-derived exosomes where a statistical increment was observed at 1 µg/mL (*p* < 0.05) and ≥5 µg/mL (*p* < 0.01 for all dosages). In cerebellar neurons, exposed to U373 GBM-derived exosomes, TOS levels statistically increase at dose of 5 µg/mL (*p* < 0.05). Dosages equal to or higher than 10 µg/mL induced more significant levels of TOS (*p* < 0.01 for all dosages) (Figure 5). 

## 4. Discussion

Despite the numerous advancements regarding the pharmacological treatments of tumors [28,29] and the novel high-sensitive techniques developed for the early diagnosis of pathologies [30,31,32,33,34], glioblastoma still represents a major issue in neuro-oncology. Although some genetic and epigenetic biomarkers for the early diagnosis of GBM are under investigation [35,36,37], different clinical signs can predict the onset of advanced tumors. Indeed, many patients with GBM present deficits in cognitive functioning and epilepsy due to brain compression that could be the first indicator of disease recurrence [4,5]. Besides mechanical dysfunction due to tumor growth and brain compression, other cellular and molecular mechanisms are involved in the development of GBM-associated brain disorders. Among these, glioblastoma cells could release factors responsible for neurotoxic effects as well as immunomodulatory factors with paracrine effects towards the surrounding neurological structures directly or through cancer-derived exosomes [38,39]. Katrib and colleagues (2019) have established a strict relationship among inflammation, GBM oxidative stress, and neurological deficit by identifying a panel of genes involved in these processes [40]. Similarly, Lange and colleagues (2021) have recently highlighted a link between glioblastoma and tumor-associated epilepsy describing the processes leading to the dysregulation of the glutamatergic signaling, including imbalance of the redox system [41]. In addition, the imbalance of the brain redox status due to the presence of GBM has been also associated with the presence of parenchymal and peritumoral edema [42]. However, the identification of the specific determinants responsible for such detrimental associations between GBM and neuronal structure has not been elucidated yet.

In order to evaluate the detrimental effects of GBM-derived exosomes, this study wanted to assess the potential neurotoxic effects mediated by GBM-derived exosomes on primary neurons.

For this purpose, cerebellar neurons were treated with increasing doses of GBM-derived exosomes obtained from two different GBM cell lines and subsequently several parameters were evaluated to assess the neurotoxic potential of such exosomes. Our results showed that neuronal cell viability decreased significantly after treatment with ≥1 µg/mL or ≥5 µg/mL of T98G- and U373-derived exosomes, respectively. MTT assay thus supports the hypothesis of the neuronal toxicity mediated by GBM-derived exosomes demonstrating a dose-dependent decrease of cell viability. This detrimental effect was also observed by analyzing the levels of GSH and LDH, two of the most recognized markers of oxidative stress and cell damage. In particular, both GSH and LDH has been widely associated with cognitive impairment and neurodegeneration [43,44,45]. In this context, Backos and co-workers showed that the accumulation of 2-deoxy-D-ribose (2dDR) due to the intratumoral necrotic processes occurring in GBM can modify the function of enzymes involved in GSH production, thus leading to oxidative stress-related alterations in the brain [46]. Similarly, several studies showed that LDH levels are increased in cancer patients, including GBM where LDH seems to be involved in cancer progression and metastases [47,48]. In addition, it was demonstrated that high levels of LDH induce longevity and neurodegeneration in animal models [49].

In our study, GSH levels were significantly reduced after treatment with exosome concentrations of 50 µg/mL and 100 µg/mL, while LDH levels increased significantly with ≥5 µg/mL or ≥50 µg/mL of T98G- or U373-derived exosomes, respectively. These data suggest how the redox potential of neuronal cells is significantly affected by the neurotoxic cargo of GBM-derived exosomes. As a consequence, the impaired antioxidant abilities of neurons lead to the accumulation of cellular damages and cell death as demonstrated by the increasing levels of LDH.

To further support these preliminary findings, the analysis of Total Antioxidant Status showed a significant reduction of the antioxidant potential of cerebellar neurons after the treatment with ≥5 µg/mL of T98G-derived exosomes or ≥50 µg/mL of U373-derived exosomes. Conversely, the analysis of the Total Oxidant Status showed an oppositive trend; TOS increased significantly when cerebellar neurons were exposed with a concentration of ≥1 µg/mL or ≥5 µg/mL of T98G- or U373-derived exosomes, respectively. Noteworthy, different results were obtained by using T98G- and U373-derived exosomes at the same concentrations. A possible explanation may be related to the different molecular cargo of T98G exosomes compared to that of U373 exosomes. In this context, Spinelli and colleagues (2018) have demonstrated that different GBM cells and molecular subtypes have totally different extracellular vesicle profiles [50]. Therefore, in the near future, the molecular profiling of both T98G and U373 exosomes will be mandatory to clearly identify proteins or other molecules (ncRNA, DNA fragments, etc.) responsible for the neurotoxic effects observed in primary cerebellar neurons.

The results here obtained are in line with other studies demonstrating the effects of GBM exosomes on tumor and brain microenvironment. In particular, it was demonstrated that GBM exosomes are able to affect brain homeostasis inducing a pro-inflammatory microenvironment due to the activation of monocytes, macrophages microglia, astrocytes and endothelial cells as well as vascular alterations mediated by the growth factors pro-angiogenic cargo consisting of EGFR, VEGF, angiogenin, PDGF, coagulation factor VIIa, etc. [51,52,53].

As regards the oxidant potential of GBM-derived exosomes here demonstrated, a possible explanation may be related to the transfer of mitochondrial damages which could increase neuron damages. In particular, Guescini and colleagues first discovered that glioblastoma cells and astrocyte release exosomes carrying mitochondrial proteins and mtDNA which transfers in neuron cells have been associated with the development of Alzheimer’s disease [54,55]. Other studies have demonstrated that mitochondrial genome (mtDNA) and even whole mitochondria are mobile and can be transferred to surrounding cells through exosomes and mitochondrial-derived vesicles inducing the activation of oxidative processes [56]. These findings may explain the high levels of 8-OHdG and the alterations of TOS and TAS observed in our experiments. 

Overall, these results demonstrated that GBM-derived exosomes are able to increase the oxidative stress in cerebellar neurons through the reduction of the cellular antioxidant defenses and the increase of oxidative damages as demonstrated in our experiments. Therefore, our study further enriched the knowledge on exosome functions in brain diseases. Indeed, a plethora of studies highlighted the pivotal role of exosomes in GBM, Alzheimer’s disease, Parkinson’s disease, epilepsy, and other brain disorders, independently [38,57,58,59,60]. However, here, for the first time, an interconnection between GBM exosomes and neuronal damages responsible for neuronal disorders has been observed.

## 5. Conclusions

To the best of our knowledge, this is the first study that demonstrates the toxic effects of glioblastoma-derived exosomes in primary neurons. The results here obtained may explain the peritumoral edema and cognitive decline often observed in glioblastoma patients. Of note, the results here obtained are merely exploratory as the precise GBM exosome factors involved in the neurotoxic effects observed in cerebellar neurons are still unknown. However, our findings will pave the way to novel investigation studies aimed at evaluating the precise molecular cargo of GBM-derived exosomes. Indeed, further in vitro and in vivo functional studies are needed to identify the precise molecules responsible for neurotoxicity and to assess the potential therapeutic effects of drugs able to reduce tumor growth, edema, and cognitive decline. 

In conclusion, the identification of a neurotoxic effect of GBM exosomes suggests how exosomes can be considered as both biomarkers and targets and their characterization could significantly improve the management of GBM patients.

## Data Availability

Data are contained within the article.

## References

[1] Altinoz M.A., Ozpinar A., Ozpinar A., Hacker E., Elmaci I. (2020). Hypothesis: Could Hepatitis B vaccine act as an immune adjuvant in glioblastoma? Clues to conduct further epidemiological analyses. Int. Immunopharmacol..

[2] Wang Y.Y., Liu S.C., Yang Z., Zhang T. (2011). Impaired hippocampal synaptic plasticity in C6 glioma-bearing rats. J. Neuro-Oncol..

[3] Erturk Cetin O., Isler C., Uzan M., Ozkara C. (2017). Epilepsy-related brain tumors. Seizure.

[4] Janelsins M.C., Kohli S., Mohile S.G., Usuki K., Ahles T.A., Morrow G.R. (2011). An update on cancer- and chemotherapy-related cognitive dysfunction: Current status. Semin. Oncol..

[5] Jacobs J., Wu J.Y., Perucca P., Zelmann R., Mader M., Dubeau F., Mathern G.W., Schulze-Bonhage A., Gotman J. (2018). Removing high-frequency oscillations: A prospective multicenter study on seizure outcome. Neurology.

[6] Li J., Liu M., Gao J., Jiang Y., Wu L., Cheong Y.K., Ren G., Yang Z. (2020). AVNP2 protects against cognitive impairments induced by C6 glioma by suppressing tumour associated inflammation in rats. Brain Behav. Immun..

[7] Venugopal C., Wang X.S., Manoranjan B., McFarlane N., Nolte S., Li M., Murty N., Siu K.W., Singh S.K. (2012). GBM secretome induces transient transformation of human neural precursor cells. J. Neurooncol..

[8] Broekman M.L., Maas S.L.N., Abels E.R., Mempel T.R., Krichevsky A.M., Breakefield X.O. (2018). Multidimensional communication in the microenvirons of glioblastoma. Nat. Rev. Neurol..

[9] Lee J., Chaloner Winton Hall R. (2019). The Impact of Gliomas on Cognition and Capacity. J. Am. Acad. Psychiatry Law..

[10] Buckingham S.C., Campbell S.L., Haas B.R., Montana V., Robel S., Ogunrinu T., Sontheimer H. (2011). Glutamate release by primary brain tumors induces epileptic activity. Nat. Med..

[11] Wei Z., Batagov A.O., Schinelli S., Wang J., Wang Y., El Fatimy R., Rabinovsky R., Balaj L., Chen C.C., Hochberg F. (2017). Coding and noncoding landscape of extracellular RNA released by human glioma stem cells. Nat. Commun..

[12] Sharma K.D., Schaal D., Kore R.A., Hamzah R.N., Pandanaboina S.C., Hayar A., Griffin R.J., Srivatsan M., Reyna N.S., Xie J.Y. (2020). Glioma-derived exosomes drive the differentiation of neural stem cells to astrocytes. PLoS ONE.

[13] Koh Y.Q., Tan C.J., Toh Y.L., Sze S.K., Ho H.K., Limoli C.L., Chan A. (2020). Role of Exosomes in Cancer-Related Cognitive Impairment. Int. J. Mol. Sci..

[14] Cui L., Pierce D., Light K.E., Melchert R.B., Fu Q., Kumar K.S., Hauer-Jensen M. (2010). Sublethal total body irradiation leads to early cerebellar damage and oxidative stress. Curr. Neurovasc. Res..

[15] Amidei C., Kushner D.S. (2015). Clinical implications of motor deficits related to brain tumors†. Neurooncol. Pr..

[16] Packer R.J., Meadows A.T., Rorke L.B., Goldwein J.L., D’Angio G. (1987). Long-term sequelae of cancer treatment on the central nervous system in childhood. Med. Pediatr. Oncol..

[17] Cantelmi D., Schweizer T.A., Cusimano M.D. (2008). Role of the cerebellum in the neurocognitive sequelae of treatment of tumours of the posterior fossa: An update. Lancet Oncol..

[18] Johnstone R.M., Adam M., Hammond J.R., Orr L., Turbide C. (1987). Vesicle formation during reticulocyte maturation. Association of plasma membrane activities with released vesicles (exosomes). J. Biol. Chem..

[19] Takahashi A., Okada R., Nagao K., Kawamata Y., Hanyu A., Yoshimoto S., Takasugi M., Watanabe S., Kanemaki M.T., Obuse C. (2017). Exosomes maintain cellular homeostasis by excreting harmful DNA from cells. Nat. Commun..

[20] Aires I.D., Ribeiro-Rodrigues T., Boia R., Catarino S., Girao H., Ambrosio A.F., Santiago A.R. (2020). Exosomes derived from microglia exposed to elevated pressure amplify the neuroinflammatory response in retinal cells. Glia.

[21] D’Asti E., Chennakrishnaiah S., Lee T.H., Rak J. (2016). Extracellular Vesicles in Brain Tumor Progression. Cell. Mol. Neurobiol..

[22] Patel G.K., Khan M.A., Zubair H., Srivastava S.K., Khushman M., Singh S., Singh A.P. (2019). Comparative analysis of exosome isolation methods using culture supernatant for optimum yield, purity and downstream applications. Sci. Rep..

[23] Tang Y.T., Huang Y.Y., Zheng L., Qin S.H., Xu X.P., An T.X., Xu Y., Wu Y.S., Hu X.M. (2017). Comparison of isolation methods of exosomes and exosomal RNA from cell culture medium and serum. Int. J. Mol. Med..

[24] Zhang S., Jiang L., Hu H., Wang H., Wang X., Jiang J., Ma Y., Yang J., Hou Y., Xie D. (2020). Pretreatment of exosomes derived from hUCMSCs with TNF-α ameliorates acute liver failure by inhibiting the activation of NLRP3 in macrophage. Life Sci..

[25] Sharma P., Mesci P., Carromeu C., McClatchy D.R., Schiapparelli L., Yates J.R., Muotri A.R., Cline H.T. (2019). Exosomes regulate neurogenesis and circuit assembly. Proc. Natl. Acad. Sci. USA.

[26] Monfared H., Jahangard Y., Nikkhah M., Mirnajafi-Zadeh J., Mowla S.J. (2019). Potential Therapeutic Effects of Exosomes Packed with a miR-21-Sponge Construct in a Rat Model of Glioblastoma. Front. Oncol..

[27] Jin Y., Wang J., Li H., Gao S., Shi R., Yang D., Wang X., Wang X., Zhu L., Wang X. (2018). Extracellular Vesicles Secreted by Human Adipose-derived Stem Cells (hASCs) Improve Survival Rate of Rats with Acute Liver Failure by Releasing lncRNA H19. EBioMedicine.

[28] Christofi T., Baritaki S., Falzone L., Libra M., Zaravinos A. (2019). Current Perspectives in Cancer Immunotherapy. Cancers.

[29] Falzone L., Salomone S., Libra M. (2018). Evolution of Cancer Pharmacological Treatments at the Turn of the Third Millennium. Front. Pharmacol..

[30] Silantyev A.S., Falzone L., Libra M., Gurina O.I., Kardashova K.S., Nikolouzakis T.K., Nosyrev A.E., Sutton C.W., Mitsias P.D., Tsatsakis A. (2019). Current and Future Trends on Diagnosis and Prognosis of Glioblastoma: From Molecular Biology to Proteomics. Cells.

[31] Candido S., Lupo G., Pennisi M., Basile M.S., Anfuso C.D., Petralia M.C., Gattuso G., Vivarelli S., Spandidos D.A., Libra M. (2019). The analysis of miRNA expression profiling datasets reveals inverse microRNA patterns in glioblastoma and Alzheimer’s disease. Oncol. Rep..

[32] Tuaeva N.O., Falzone L., Porozov Y.B., Nosyrev A.E., Trukhan V.M., Kovatsi L., Spandidos D.A., Drakoulis N., Kalogeraki A., Mamoulakis C. (2019). Translational Application of Circulating DNA in Oncology: Review of the Last Decades Achievements. Cells.

[33] Certo F., Altieri R., Maione M., Schonauer C., Sortino G., Fiumanò G., Tirrò E., Massimino M., Broggi G., Vigneri P. (2021). FLAIRectomy in Supramarginal Resection of Glioblastoma Correlates with Clinical Outcome and Survival Analysis: Prospective, Single Institution, Case Series. Oper. Neurosurg..

[34] Falzone L., Gattuso G., Tsatsakis A., Spandidos D.A., Libra M. (2021). Current and innovative methods for the diagnosis of COVID-19 infection (Review). Int. J. Mol. Med..

[35] Barbagallo D., Caponnetto A., Barbagallo C., Battaglia R., Mirabella F., Brex D., Stella M., Broggi G., Altieri R., Certo F. (2021). The GAUGAA Motif Is Responsible for the Binding between circSMARCA5 and SRSF1 and Related Downstream Effects on Glioblastoma Multiforme Cell Migration and Angiogenic Potential. Int. J. Mol. Sci..

[36] Broggi G., Salvatorelli L., Barbagallo D., Certo F., Altieri R., Tirrò E., Massimino M., Vigneri P., Guadagno E., Maugeri G. (2021). Diagnostic Utility of the Immunohistochemical Expression of Serine and Arginine Rich Splicing Factor 1 (SRSF1) in the Differential Diagnosis of Adult Gliomas. Cancers.

[37] Stella M., Falzone L., Caponnetto A., Gattuso G., Barbagallo C., Battaglia R., Mirabella F., Broggi G., Altieri R., Certo F. (2021). Serum Extracellular Vesicle-Derived circHIPK3 and circSMARCA5 Are Two Novel Diagnostic Biomarkers for Glioblastoma Multiforme. Pharmaceuticals.

[38] Balasa A., Serban G., Chinezu R., Hurghis C., Tamas F., Manu D. (2020). The Involvement of Exosomes in Glioblastoma Development, Diagnosis, Prognosis, and Treatment. Brain Sci..

[39] Ciregia F., Urbani A., Palmisano G. (2017). Extracellular Vesicles in Brain Tumors and Neurodegenerative Diseases. Front. Mol. Neurosci..

[40] Katrib A., Jeong H.H., Fransen N.L., Henzel K.S., Miller J.A. (2019). An Inflammatory Landscape for Preoperative Neurologic Deficits in Glioblastoma. Front. Genet..

[41] Lange F., Hörnschemeyer J., Kirschstein T. (2021). Glutamatergic Mechanisms in Glioblastoma and Tumor-Associated Epilepsy. Cells.

[42] Șovrea A.S., Boșca B., Melincovici C.S., Constantin A.M., Crintea A., Mărginean M., Dronca E., Jianu M.E., Suflețel R., Gonciar D. (2022). Multiple Faces of the Glioblastoma Microenvironment. Int. J. Mol. Sci..

[43] Wefel J.S., Noll K.R., Scheurer M.E. (2016). Neurocognitive functioning and genetic variation in patients with primary brain tumours. Lancet Oncol..

[44] Facecchia K., Fochesato L.A., Ray S.D., Stohs S.J., Pandey S. (2011). Oxidative toxicity in neurodegenerative diseases: Role of mitochondrial dysfunction and therapeutic strategies. J. Toxicol..

[45] Ross J.M., Öberg J., Brené S., Coppotelli G., Terzioglu M., Pernold K., Goiny M., Sitnikov R., Kehr J., Trifunovic A. (2010). High brain lactate is a hallmark of aging and caused by a shift in the lactate dehydrogenase A/B ratio. Proc. Natl. Acad. Sci. USA.

[46] Backos D.S., Fritz K.S., McArthur D.G., Kepa J.K., Donson A.M., Petersen D.R., Foreman N.K., Franklin C.C., Reigan P. (2013). Glycation of glutamate cysteine ligase by 2-deoxy-d-ribose and its potential impact on chemoresistance in glioblastoma. Neurochem. Res..

[47] Valvona C.J., Fillmore H.L., Nunn P.B., Pilkington G.J. (2016). The Regulation and Function of Lactate Dehydrogenase A: Therapeutic Potential in Brain Tumor. Brain Pathol..

[48] Daniele S., Giacomelli C., Zappelli E., Granchi C., Trincavelli M.L., Minutolo F., Martini C. (2015). Lactate dehydrogenase-A inhibition induces human glioblastoma multiforme stem cell differentiation and death. Sci. Rep..

[49] Long D.M., Frame A.K., Reardon P.N., Cumming R.C., Hendrix D.A., Kretzschmar D., Giebultowicz J.M. (2020). Lactate dehydrogenase expression modulates longevity and neurodegeneration in Drosophila melanogaster. Aging.

[50] Spinelli C., Montermini L., Meehan B., Brisson A.R., Tan S., Choi D., Nakano I., Rak J. (2018). Molecular subtypes and differentiation programmes of glioma stem cells as determinants of extracellular vesicle profiles and endothelial cell-stimulating activities. J. Extracell. Vesicles.

[51] Spinelli C., Tawil N., Adnani L., Rak J., Choi D. (2021). Extracellular Vesicle Mediated Vascular Pathology in Glioblastoma. Subcell. Biochem..

[52] Matarredona E.R., Pastor A.M. (2019). Extracellular Vesicle-Mediated Communication between the Glioblastoma and Its Microenvironment. Cells.

[53] Yekula A., Yekula A., Muralidharan K., Kang K., Carter B.S., Balaj L. (2020). Extracellular Vesicles in Glioblastoma Tumor Microenvironment. Front. Immunol..

[54] Guescini M., Genedani S., Stocchi V., Agnati L.F. (2010). Astrocytes and Glioblastoma cells release exosomes carrying mtDNA. J. Neural. Transm..

[55] Agnati L.F., Guidolin D., Baluska F., Leo G., Barlow P.W., Carone C., Genedani S. (2010). A new hypothesis of pathogenesis based on the divorce between mitochondria and their host cells: Possible relevance for Alzheimer’s disease. Curr. Alzheimer Res..

[56] Singh B., Modica-Napolitano J.S., Singh K.K. (2017). Defining the momiome: Promiscuous information transfer by mobile mitochondria and the mitochondrial genome. Semin. Cancer Biol..

[57] Jin Q., Wu P., Zhou X., Qian H., Xu W. (2021). Extracellular Vesicles: Novel Roles in Neurological Disorders. Stem. Cells Int..

[58] Jiang L., Dong H., Cao H., Ji X., Luan S., Liu J. (2019). Exosomes in Pathogenesis, Diagnosis, and Treatment of Alzheimer’s Disease. Med. Sci. Monit..

[59] Porro C., Panaro M.A., Lofrumento D.D., Hasalla E., Trotta T. (2019). The multiple roles of exosomes in Parkinson’s disease: An overview. Immunopharmacol. Immunotoxicol..

[60] Karttunen J., Heiskanen M., Lipponen A., Poulsen D., Pitkänen A. (2019). Extracellular Vesicles as Diagnostics and Therapeutics for Structural Epilepsies. Int. J. Mol. Sci..

